# Supplementary material for: Van der Waals thin films of WTe2 for natural hyperbolic plasmonic surfaces
Source: Nat Commun. 2020 Mar 3;11:1158. doi: 10.1038/s41467-020-15001-9 (PMC7054419; doi:10.1038/s41467-020-15001-9)
Supplement: Supplementary file 1 — Supplementary Information [file 41467_2020_15001_MOESM1_ESM.pdf]

Supplementary Information for

Van der Waals thin films of WTe_2 for natural hyperbolic plasmonic surfaces

Chong Wang et al.

Supplementary Note I: Derivation of the Relation between the Longitudinal Conductivity and the Dielectric Function

In this note, we will derive the relation between the longitudinal conductivity and the dielectric function within the random phase approximation (RPA). The final result, however, is general[1]. The RPA dielectric function can be written in terms of the Lindhard polarization as:

$$\varepsilon^{\mathrm{RPA}}(\mathbf{q},\omega)=\varepsilon_r - v_q P(\mathbf{q},\omega) \tag{1}$$

where, $v_q = e^2/2\varepsilon_0 q$ stands for the Fourier transform of the Coulomb interaction in 2D case, ε_r is the relative permittivity of the medium, and $P(\mathbf{q},\omega)$ is the 2D polarizability (the density-density correlation function) given by the bare bubble diagram[2]. On the other hand, the conductivity is related to the current-current correlation function. In the linear response theory framework, the longitudinal conductivity is given by:

$$\sigma_L = \frac{i}{\omega}\left(\frac{ne^2}{m^*}+\Pi^L\right) \tag{2}$$

where, Π^L is the longitudinal part of the current-current correlation function, n is the carrier density and m^* is the effective mass of electrons. The conductivity has two contributions: the first from the background charge density; the second form the retarded Green's function (Π^L). From the usual charge continuity equation, $\partial_t \rho + \nabla \cdot J = 0$, we can have the relation between the density correlation and current correlation functions as following:

$$\omega^2 \Pi^{00} = \frac{ne^2}{m^*} q^2 + \Pi^L q^2 \tag{3}$$

By combining Supplementary Eq. 2 and Supplementary Eq. 3, one can obtain the relation between the density-density correlation function and the longitudinal conductivity:

$$\sigma_L = \frac{i\omega}{q^2}\Pi^{00} \tag{4}$$

Because $e^2 P \equiv \Pi^{00}$, by using Supplementary Eq. 4 and Supplementary Eq. 1, we can derive the expression relating the dielectric function to the longitudinal conductivity as shown in the main text (Eq. 3 of the main text).

Supplementary Note II: Plasmon Dispersion under the Influence of both Intraband and Interband Transitions

In this note, we will derive the plasmon dispersion equation $\omega = \omega(q)$ by considering both the intraband and interband transitions in the optical conductivity.

By substituting the conductivity (Eq. 1 in the main text) into the dielectric function (Eq.3 in the main text), we can have:

$$\varepsilon_{jj} = \varepsilon_r + \frac{i}{\varepsilon_0 \omega}\left(i\frac{D_{jj}}{\pi(\omega + i\Gamma)} + i\frac{\omega S_{jj}}{\pi\left(\omega^2 - {\omega_{\mathrm{b}}}^2 + i\omega\eta\right)} \right)\frac{q}{2} \tag{5}$$

For simplify, the scattering width Γ and η are set to zero (losses are small in our system). Since plasmon dispersion can be obtained from the zeros of the dielectric function, we have:

$$2\pi\varepsilon_r\varepsilon_0\omega^4 - (2\pi\varepsilon_r\varepsilon_0\omega_{\mathrm{b}}^2 + qD_{jj} + qS_{jj})\omega^2 + qD_{jj}\omega_{\mathrm{b}}^2 = 0 \tag{6}$$

This gives rise to two branches of plasmon:

$$\omega_{\pm}^{jj} = \frac{\sqrt{2\pi\varepsilon_r\varepsilon_0\omega_{\mathrm{b}}^2 + qD_{jj} + qS_{jj} \pm \sqrt{\left(2\pi\varepsilon_r\varepsilon_0\omega_{\mathrm{b}}^2 + qD_{jj} + qS_{jj}\right)^2 - 4\cdot 2\pi\varepsilon_r\varepsilon_0 \cdot qD_{jj}\omega_{\mathrm{b}}^2}}}{4\pi\varepsilon_r\varepsilon_0} \tag{7}$$

The upper branch is associated with the interband transition and the lower branch starts form zero at low q, which is the normal plasmon we focus on. When the wave vector increases, the plasmon of the lower branch approaches to a constant energy where the imaginary part of the conductivity is zero, since we have $\mathrm{Im}\,\sigma/2\varepsilon_0\varepsilon_r\omega = 1/q$. The upper boundary of the lower branch can be deduced by Supplementary Eq. 6, from which we can have:

$$\frac{D_{jj}}{\pi\omega}+\frac{\omega S_{jj}}{\pi(\omega^2-\omega_{\mathrm{b}}^2)}=\frac{2\varepsilon_r\varepsilon_0\omega}{q} \tag{8}$$

When $q\to+\infty$, the right hand side of the equation equals zero, and the energy of the upper boundary of the lower branch can be derived as:

$$\omega_{\text{boundary}}^{jj}=\sqrt{\frac{D_{jj}}{D_{jj}+S_{jj}}}\omega_{\mathrm{b}} \tag{9}$$

It shows that the hyperbolic frequency range is determined by the anisotropy of intraband and interband transitions along the two axes. In WTe_2 film, the Drude weight $D_{aa}>D_{bb}$, while the interband transition has opposite anisotropy of $S_{aa}<S_{bb}$, which tends to facilitate the formation of a relatively broad hyperbolic frequency range. From the loss function fitting, we have $D_{aa}/S_{aa}=1.875$ and $D_{bb}/S_{bb}=0.556$ ($\omega_b=710$ cm^{-1}). These parameters give the lower and upper boundaries of the hyperbolic regime of 424 cm^{-1} and 573 cm^{-1}, respectively, which is consistent with the range (429 cm^{-1} to 632 cm^{-1}) given by the imaginary parts of conductivity calculated from the loss function (Fig. 4b in the main text). The difference is due to the neglect of the scattering width in the above analysis. The interband transition width is relatively large (170 cm^{-1}) and has certain effect on the plasmon asymptotic frequencies.

Supplementary Note III: Iso-frequency Contour Equation of 2D Plasmons in Wave Vector Space

The inserts of Fig. 4a in the main text display the iso-frequency contours of plasmons in wave vector space, which are plotted based on the plasmon dispersion relation in 2D materials. Assuming low loss plasmons with $\lambda_{\mathrm{p}}<\lambda$, where λ_{p} and λ are the wavelengths of plasmon and light in vacuum respectively, the simplified plasmon dispersion in in-plane

anisotropic 2D materials is given by[3]

$$2\sqrt{k_x^2+k_y^2}\left(\frac{\varepsilon_0}{\sigma''_{xx}\sigma''_{yy}}-\frac{\mu_0}{4}\right)\omega=\frac{k_x^2}{\sigma''_{xx}}+\frac{k_y^2}{\sigma''_{yy}} \tag{10}$$

where k_x and k_y are the in-plane wave vectors, σ''_{jj} (j = x, y) are the imaginary parts of the two principle components of the conductivity, and ε_0, μ_0 are the vacuum permittivity and permeability, respectively. Here, the 2D material is assumed to be in vacuum, with the relative permittivity of surrounding environment of $\varepsilon_r = 1$. The inserts in Fig. 4a of the main text are plotted with $\sigma''_{xx}/\sigma''_{yy}$ = 2 and -2 for the elliptic and hyperbolic regimes, respectively. Supplementary Equation 10 indicates that, for hyperbolic plasmons, the iso-frequency contour is a hyperbola, existing in the spectral range where $\sigma''_{xx}\sigma''_{yy} < 0$. On the other hand, the regime with $\sigma''_{xx}\sigma''_{yy} > 0$ supports purely anisotropic plasmons, with elliptic iso-frequency contour in ***k***-space.

Supplementary Note IV: Discussion on the Temperature Dependence of the Plasmon Frequency

The plasmon frequencies of WTe_2 films are tunable by temperature as shown in Fig. 2c of the main text and Supplementary Fig. 3. One possible reason is the change of effective mass, which should be different along the two axes, resulting in the decreased effective mass ratio as shown in Fig. 2e of the main text. Another reason is the increase of carrier density with temperature, as a result of thermal excitation. In Weyl semimetals like $Eu_2Ir_2O_7$, temperature dependence of the Drude spectral weight is observed at low temperature, which is attributed to the carrier density change induced by temperature because of the relatively low Fermi energy[4]. This is in contrast to far IR plasmon in graphene, whose frequency is nearly independent of

temperature due to the high doping level (typically ~300 meV). The calculated Fermi energy in undoped WTe_2 is about 55 meV away from the Weyl nodes[5]. However, the value is possible to be tuned further close to the Weyl nodes through the unintentional residual doping during the crystal synthesis, making the carrier density even more strongly temperature dependent.

It should be noted that, a singularity in the temperature dependence of plasmon frequency, as shown in Fig. 2c of the main text, can be observed along both axes at around 150 K, above which the plasmon frequency has a sudden change. This is reproducible in other disk arrays of WTe_2 (See Supplementary Fig. 5). Since plasmons are the collective modes of electrons, this singularity is possibly related to the change of the electronic structure. The temperature-induced Lifshitz transition[6] reported at about 160 K, which represents the complete disappearance of the hole pockets, may explain the observed singularity.

Supplementary Note V: Coupled Oscillator Model.

The intensity evolution of plasmons can be phenomenologically fitted by the coupled oscillator model[7], as shown in Fig. 4c in the main text. Here, the loss function cannot give a full explanation of the plasmon spectral weight quantitatively, since it yields a direct analogy to the electron energy loss spectroscopy (EELS) experimental spectra. Supplementary Fig. 8a shows the schematic of two coupled mechanical oscillators, with the plasmonic oscillation and bound state represented by particle 1 with mass m_1 and particle 2 with mass m_2, respectively. The springs with spring constants k_1 and k_2 are attached to a wall, which determine the intrinsic oscillation frequencies. Another spring with constant K is attached between the two particles, which is responsible for the coupling between them. When a harmonic force $F_s = Fe^{-i\omega_s t}$, with

ω_s being the driving frequency, is applied to particle 1, the equations of motion read:

$$\ddot{x}_1 + \gamma_1 \dot{x}_1 + \omega_1 x_1^2 - \Omega^2 x_2 = \frac{F}{m}\exp\left(-i\omega_s t\right)$$

$$\ddot{x}_2 + \gamma_2 \dot{x}_2 + \omega_2 x_2^2 - \Omega^2 x_1 = 0 \qquad (11)$$

where x_1 and x_2 are the displacements, ω_1 and ω_2 are the frequencies of the two oscillators without coupling, γ_1 and γ_2 are the damping rates, and $\Omega = \sqrt{K/m}$ is a frequency associated with the coupling constant. For simplify, we set $m_1 = m_2 = m$. The average power absorbed by oscillator 1 as a function of the driving frequency can be written as:

$$P\left(\omega_s\right) = \frac{2\pi i F^2 \omega_s \left(\omega_2^2 - \omega_s^2 - i\gamma_2\omega_2\right)}{m\left[\left(\omega_1^2 - \omega_s^2 - i\gamma_1\omega_1\right)\left(\omega_2^2 - \omega_s^2 - i\gamma_2\omega_2\right) - \Omega^4\right]} \qquad (12)$$

Here, ω_1 and ω_2 are determined by the plasmon oscillation of free carriers and the bound state of interband transitions without intraband and interband coupling, respectively. Thus, we have $\omega_1 = A\sqrt{q}$, with the parameter A along *a* (*b*) axis determined by the plasmons of free carriers along *a* (*b*) axis as shown by the dashed solid line in Fig. 4a of the main text; ω_2 is set to 710 cm^{-1}; γ_1 and γ_2 are 60 cm^{-1} and 170 cm^{-1}, respectively. At a given wave vector, the value of Ω for plasmons along *a* (*b*) axis can be determined by fitting the lower peak in the real part of spectra of $P\left(\omega_s\right)$ to the plasmon frequency at the same *q* along *a* (*b*) axis fitted by the loss function as shown in Fig. 4a of the main text. The fitting results of Ω along *a* and *b* axes are shown in Supplementary Fig. 8e. It shows that Ω increases with increasing wave vector, implying the power absorption of particle 1 decreases at high wave vector due to the stronger coupling between the two mechanical oscillators. This is consistent with the case of plasmon in WTe_2 film, where the spectral weight of plasmon is severely reduced near the boundary of the hyperbolic regime due to the coupling between the plasmon and interband transitions. The plasmon dispersion along *a* and *b* axes can be calculated from $P\left(\omega_s, q\right)$ with

the fitted Ω along *a* and *b* axes, as shown in Supplementary Fig. 8b, which gives a well description of the measured plasmon modes. Here the mass ratio between *a* and *b* axis is set to 2.3. The plasmon intensity evolution in Fig. 4c of the main text is extracted from the fitting results in Supplementary Fig. 8b.

Because of the coupling between the two oscillators, the dissipation of the power absorption of particle 1 is partitioned by two branches, as shown in Supplementary Fig. 8c. The upper and lower branches represent the plasmonic oscillation induced by interband transition (interband plasmon) and intraband transition (intraband plasmon). As the wave vector increases, the intensity of the intraband plasmon decreases, while the interband plasmon gains in spectral weight. This leads to an increasing intensity ratio between the interband plasmon and intraband plasmon, as shown in Supplementary Fig. 8d.

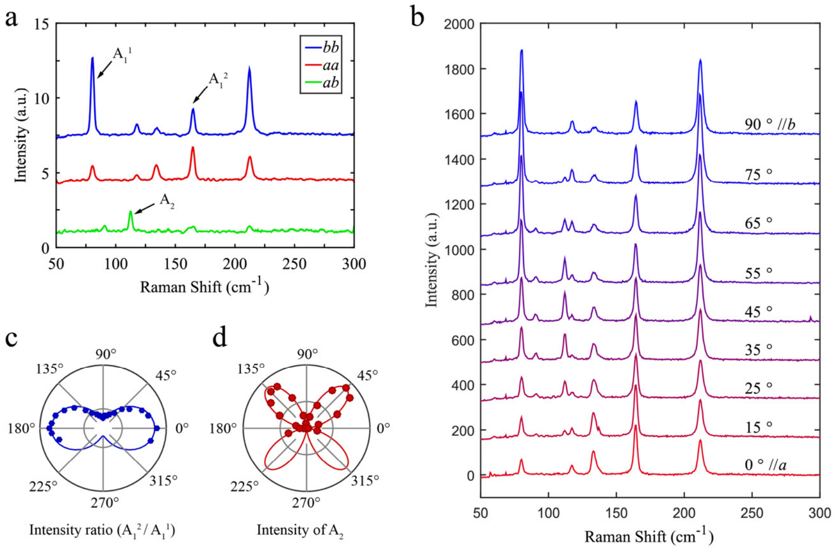

Supplementary Figure 1 | Polarized Raman spectra of WTe_2 thin films. Raman spectra of the WTe_2 film shown in Fig. 1c in the main text. Raman measurements were performed using a Horiba HR LabRam system. A 100x objective lens was used to focus the 514 nm laser. Attenuation of the incident laser was made to reduce the power below 0.5 mW to minimize sample damage. **(a)** Raman spectra in the parallel (*aa* and *bb*) and cross (*ab*) polarization configurations. Here, A_1^1 at around 81 cm^{-1} and A_1^2 modes at around 165 cm^{-1} can be only observed in the parallel scheme, while A_2 mode at around 112 cm^{-1} exists in the cross scheme, consistent with the Raman selection rules determined by the crystal symmetry[8]. Despite of the same number of Raman active phonon modes in *aa* and *bb* configurations, the intensity ratios between A_1^1 and A_1^2 modes are quite different. **(b)** The angle-dependent Raman intensity spectra of WTe_2 film, obtained by rotating the sample in the parallel polarization configuration. We can see the intensity of A_1^1 (A_1^2) increases (decreases) with increasing angle, while the A_2 mode has the largest intensity at angle of 45 degrees. Thus, it is convenient to distinguish the crystal orientation in WTe_2 by the angle dependence of the intensity ratio of A_1^2 / A_1^1 **(c)** and the intensity of A_2 mode **(d)**.

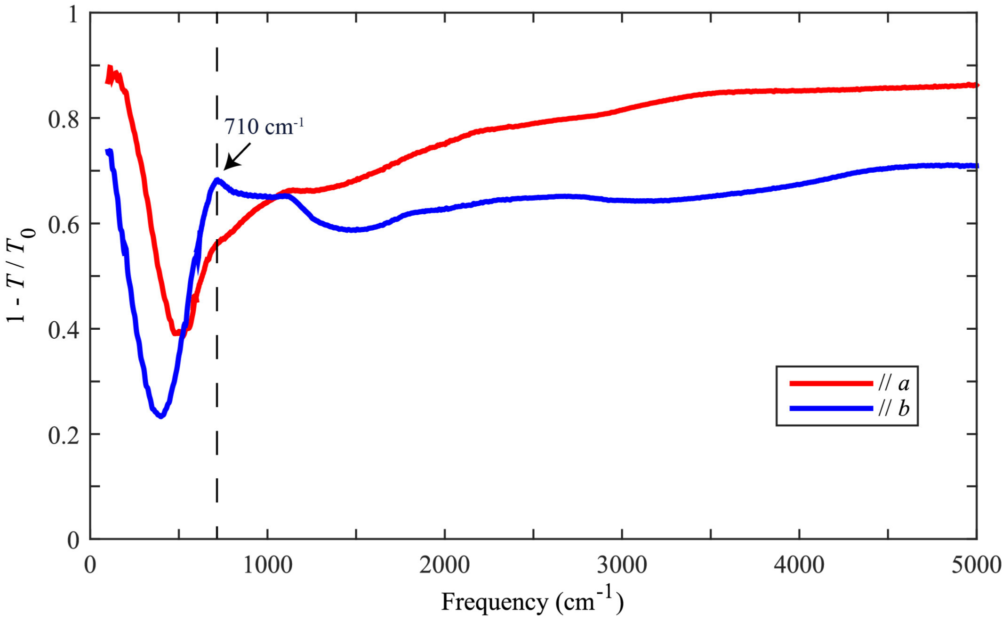

Supplementary Figure 2 | Extinction spectra of a WTe_2 film on CVD-diamond in mid- and far-IR regimes. Film thickness is about 100 nm. Temperature is at 10 K. At low energy where the Drude response dominates, the spectrum has larger absorption with the polarization along *a* axis than that along *b* axis. However, the bound state at around 710 cm^{-1} has opposite anisotropy, for which the spectrum along *b* axis is more intense than that along *a* axis.

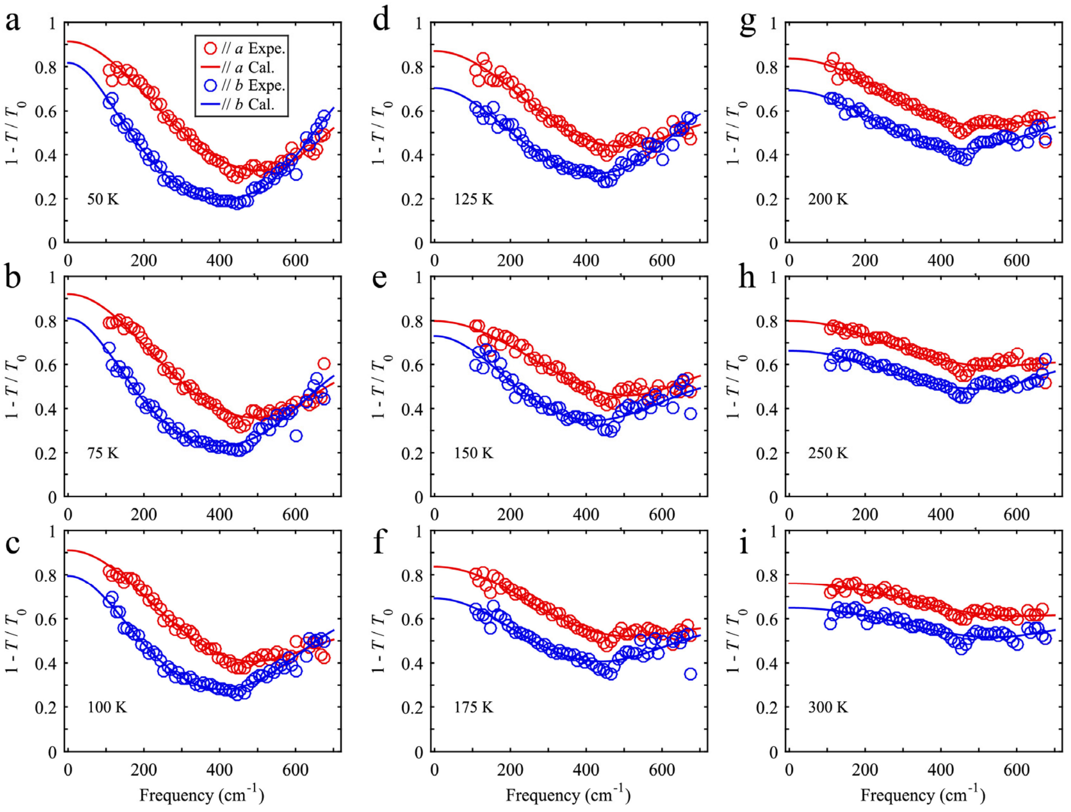

Supplementary Figure 3 | Temperature dependence of the Drude response in the exfoliated WTe_2 film. (a) to **(i)** Extinction spectra of the WTe_2 thin film in Fig. 1c in the main text with polarization along *a* and *b* axes at different temperature. Solid curves are corresponding fittings, with the Drude response and interband transition taken into account. A clear decrease of the scattering width of the Drude response can be observed when temperature is cooled down to 50 K from room temperature.

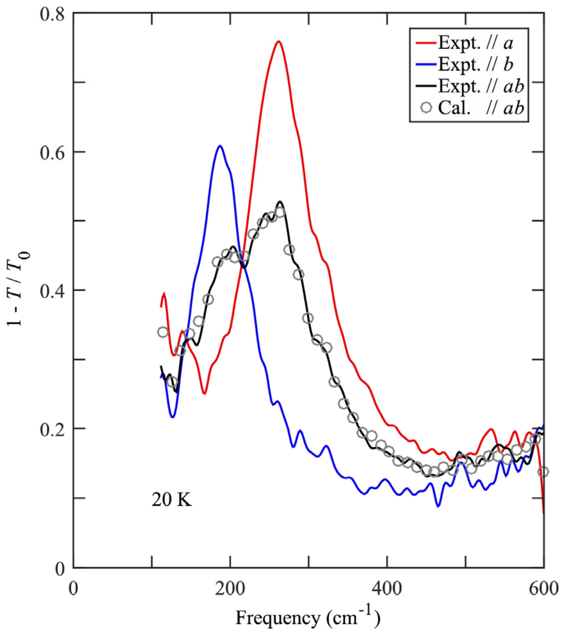

Supplementary Figure 4 | Polarization dependence of plasmons in a disk array. Plasmon resonance modes of a disk array in Fig. 2 of the main text are shown with polarization along *a*, *b* and *ab* directions. The open circles are calculated by summing the spectra along *a* and *b* axes with weight factors of $\cos^2\theta$ and $\sin^2\theta$ ($\theta = 45^\circ$), respectively. It is noted that, due to lattice symmetry, only plasmon modes along two principle axes are supported in the disk cavities. That is why when light is polarized along the middle of the two optical axes, the resonance mode exhibits two-peak structure (black line) and no new mode appears. This spectrum can be perfectly reproduced by averaging the plasmon spectra along *a* and *b* axes (open circles).

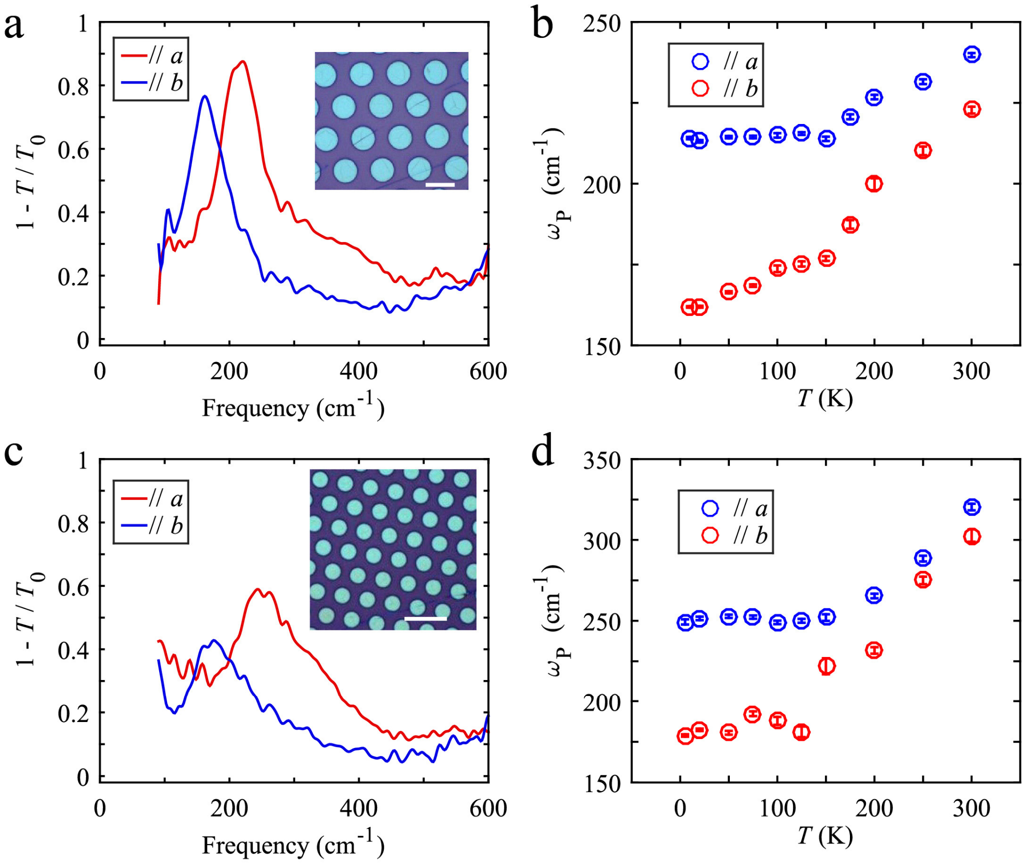

Supplementary Figure 5 | Temperature dependence of plasmon frequencies in two sets of WTe_2 disk arrays. **(a)** and **(c)** Extinction spectra of plasmon resonance modes along *a* and *b* axes in the disk arrays fabricated on WTe_2 film with thickness of about 100 nm **(a)** and 45 nm **(c).** Temperature is at 10 K. Inserts: Optical microscope images of the disk arrays with diameter of 8 μm **(a)** and 4 μm **(c)**. Scale bar is 10 μm. The calculated effective mass ratio is about 1.8 and 1.95 for **(a)** and **(c)**, respectively. The upturn of the spectra at the low energy side in **(c)** is attributed to the Drude response from the unpatterned film outside of the patterned area. **(b)** and **(d)** Temperature dependence of the fitted plasmon frequencies of the disk arrays shown in **(a)** and **(c)**. Singularities can be found at around 150 K along both axes in **(b)** and **(d)**, consistent with the results in Fig. 2c of the main text. Error bars are defined from fittings.

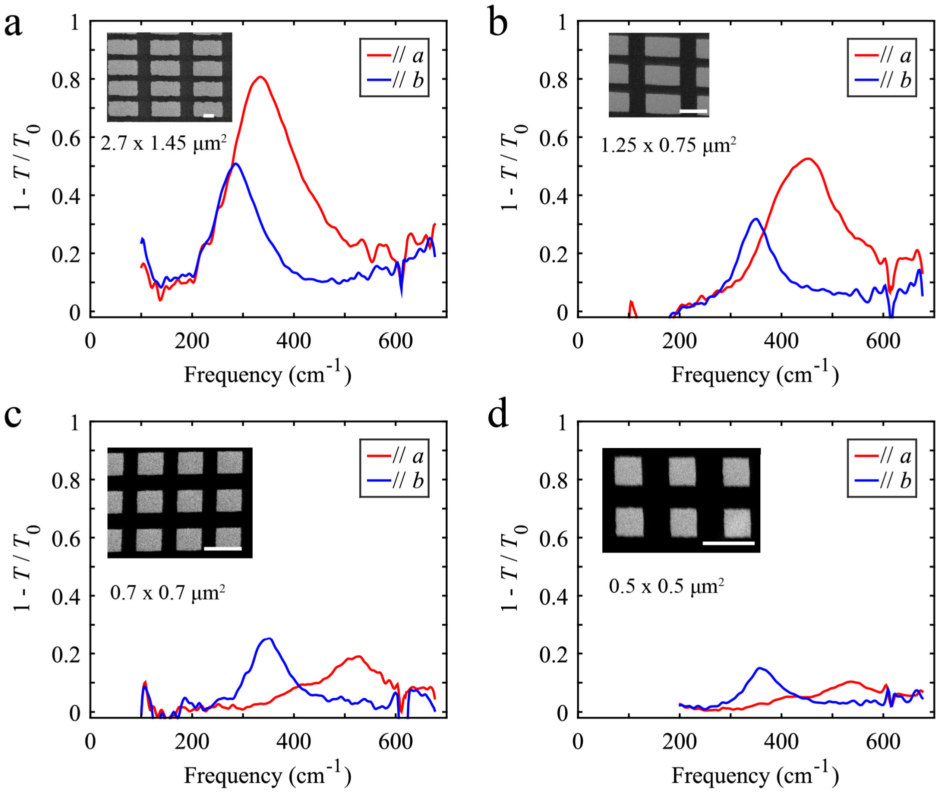

Supplementary Figure 6 | Extinction spectra along *a* and *b* axes in the WTe_2 rectangle/square arrays with different sizes. (a) to **(d)** The inserts show the SEM images of the rectangle/square arrays, with *a* axis pointing along horizontal direction. Scale bar is 1 μm. The plasmon modes can be observed with lower intensity in smaller rectangle/square arrays. This is because: first, the filling factors are decreased in small rectangle/square arrays; second, the interaction between the plasmon mode and interband transitions transfers the spectral weight of plasmon near hyperbolic frequency regime to interband plasmons at higher energies. A small peak can be observed or fitted at about 400 cm^{-1} in panels **(b)**, **(c)** and **(d)**, the intensity of which is small compared to the main peak. In our measurements, this peak always sits at 400 cm^{-1} and only appears when the main peak is larger than 400 cm^{-1}. Since 400 cm^{-1} is near the energy for topological transition from the elliptic to the hyperbolic regime, this small peak might come from some higher order modes associated with the topological transition.

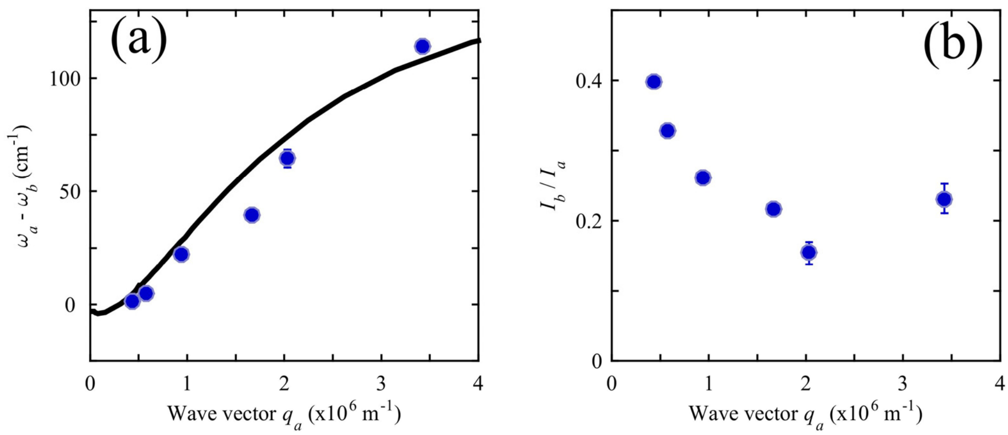

Supplementary Figure 7 | Analysis of the two trends of the plasmon spectra in Fig. 3a of the main text. (a) and **(b)** Frequency difference and intensity ratio between plasmonic resonance modes along the two principle axes in rectangle arrays in Fig. 3a of the main text, as a function of the wave vector *q* along *a* axis ($q = \pi / L_a$). The black curve in **(a)** is fitted by the loss function described in the main text. The plasmon intensity along *b* axis has a larger reduction rate, leading to a decreasing intensity ratio between *b* and *a* axes in **(b)**. Error bars are defined from fittings.

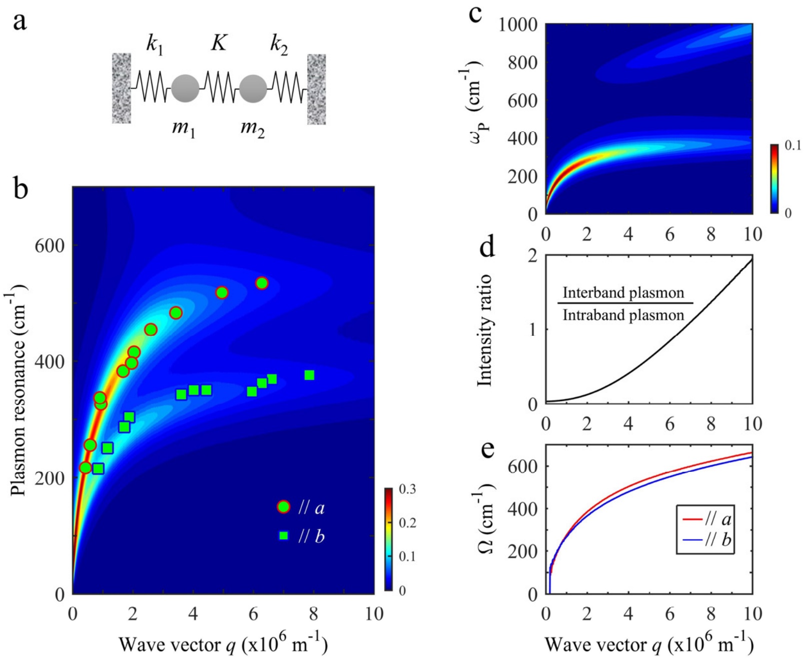

Supplementary Figure 8 | Fitting the plasmon dispersion by coupled oscillator model. (a) Schematic of the two coupled mechanical oscillators. K , k_1 and k_2 are the spring constants. m_1 and m_2 are the masses. **(b)** Plasmon frequencies of rectangle arrays on CVD-grown diamond substrates as a function of wave vector $q = \pi/L$, with $L = L_a$ and L_b for polarization along *a* and *b* axes, respectively. The dispersion fitted by the coupled oscillator model is displayed as the contour plot, with the pseudo-color representing the power absorption of oscillator 1 (plasmon mode) from the driving force. Here, the fitting results along two axes are shown in one map. **(c)** Plasmon dispersion along *b* axis in a wider frequency range fitted by the coupled oscillator model. Two branches can be observed, which are attributed to the interband plasmon (upper branch) and intraband plasmon (lower branch). **(d)** The intensity ratio between interband plasmon and intraband plasmon along *b* axis in **(c)** as a function of the wave vector. **(e)** Coupling parameter Ω extracted from the dispersion fitting in **(b)** as a function of the wave vector, which also determines the plasmon mode intensity shown in Fig. 4c of the main text.

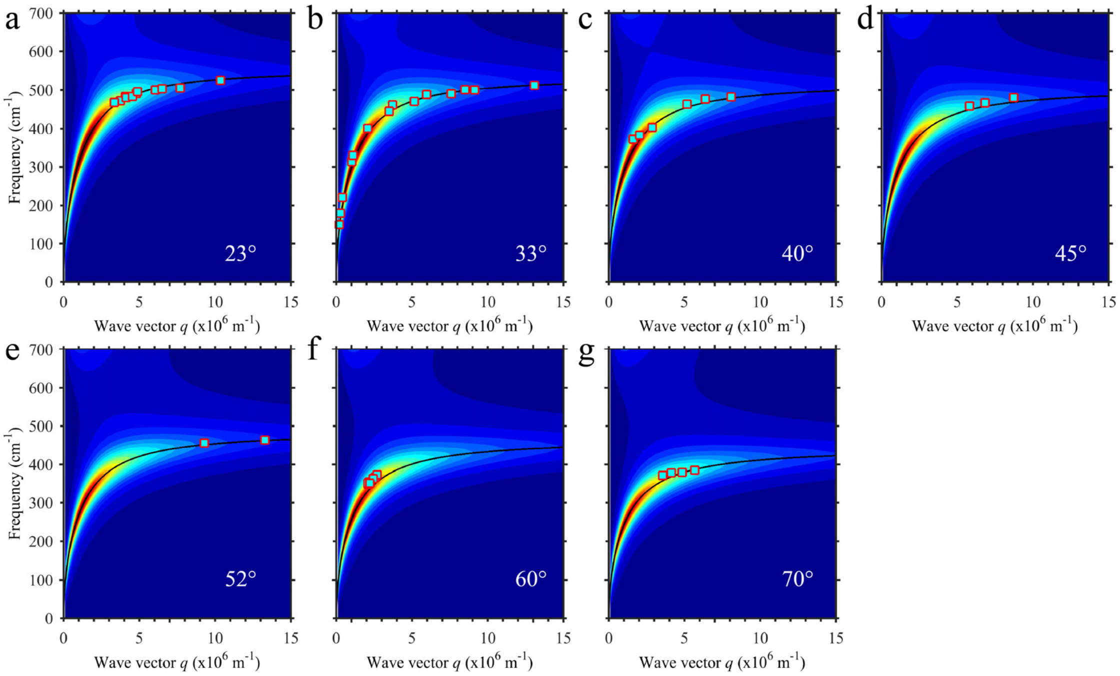

Supplementary Figure 9 | Plasmon dispersion along wave vectors away from principle axes. (a) to **(g)** Plasmon resonance peaks in skew ribbon arrays as a function of wave vector with $q = \pi / L$ (L is the ribbon width). The calculated loss function is displayed as a pseudo-color map (see methods in the main text). The solid black curves represent the fitted dispersion (see methods in the main text).

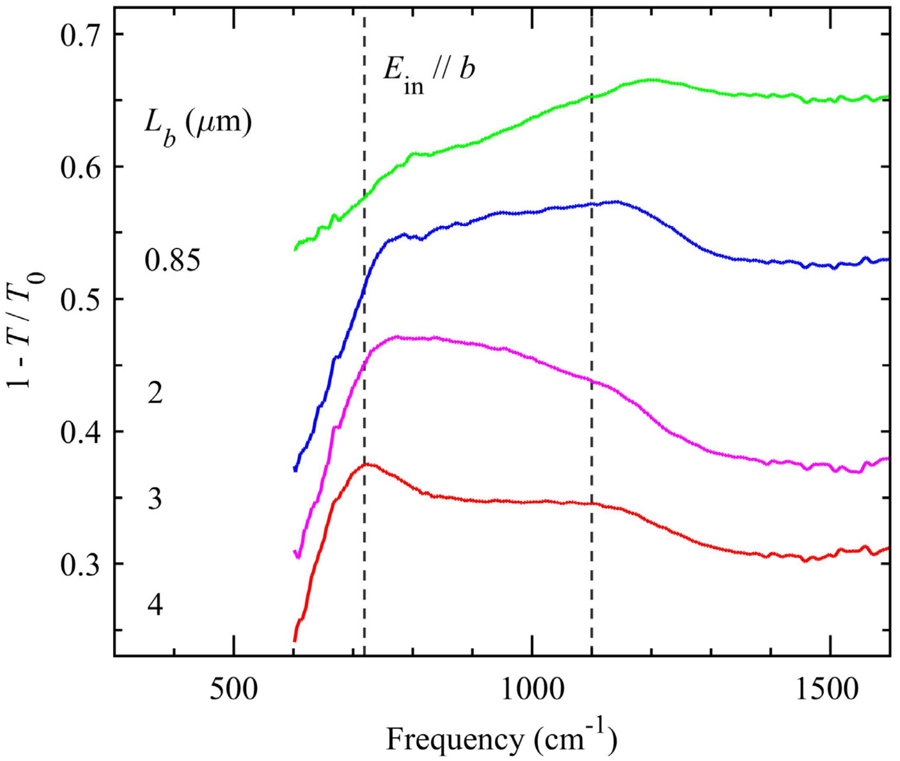

Supplementary Figure 10 | Extinction spectra (vertical offset for clarity) along *b* axis of rectangle arrays with different length of L_b in the mid-infrared frequency range. All the arrays have the same film thickness about 100 nm.

Nominal size $L_a \times L_b$ (μm^2)	Real value L_a (μm)	Real value L_b (μm)	Film thickness (nm)
8 x 4	8.1	4.1	110
6 x 3	6.0	3.0	110
4 x 2	3.7	1.85	110
2 x 1	1.9	0.89	102
1.7 x 0.85	1.7	0.89	110
1 x 0.5	1.1	0.57	120
2.7 x 1.45	2.79	1.47	80
1.25 x 0.75	1.25	0.73	103
0.5 x 0.5	0.55	0.55	110
0.7 x 0.7	0.78	0.65	123
1.8 x 0.45	1.85	0.46	115

Supplementary Table 1| Summary of the rectangle size and film thickness for different rectangle arrays discussed in the main text. Because of the relatively large film thickness, lateral etching exists when we etch the nanostructures, which makes the real value of the structure size smaller than the designed one. In order to compensate the length decrease during the etching process and obtain the desired structures (nominal sizes listed in the table), the rectangle length is designed a little longer than the desired structure according to the lateral etching rate. The obtained real lengths are summarized in the table, which are managed to be within 10% of uncertainty of the nominal values. The corresponding aspect ratio L_a/L_b is managed to be 2 ± 0.15 for the rectangles in Fig. 3a of the main text.

Supplementary References:

1 Gonçalves, P. A. D., Peres, N. M. *An Introduction to Graphene Plasmonics* (World Scientific, 2016).

2 Mahan, G. D. *Many Particle Physics 3rd*. (Springer Science & Business Media, 2000).

3 Nemilentsau, A., Low, T., Hanson, G. Anisotropic 2D Materials for Tunable Hyperbolic Plasmonics. *Phys. Rev. Lett.* **116,** 066804 (2016).

4 Sushkov, A. B.*, et al.* Optical evidence for a Weyl semimetal state in pyrochlore $Eu_2Ir_2O_7$. *Phys. Rev. B* **92,** 241108 (2015).

5 Soluyanov, A. A.*, et al.* Type-II Weyl semimetals. *Nature* **527,** 495-498 (2015).

6 Wu, Y.*, et al.* Temperature-Induced Lifshitz Transition in WTe_2. *Phys. Rev. Lett.* **115,** 166602 (2015).

7 Garrido Alzar, C. L., Martinez, M. A. G., Nussenzveig, P. Classical analog of electromagnetically induced transparency. *Am. J. Phys.* **70,** 37-41 (2002).

8 Song, Q.*, et al.* The polarization-dependent anisotropic Raman response of few-layer and bulk WTe_2 under different excitation wavelengths. *RSC Adv.* **6,** 103830-103837 (2016).
